# Tolerance Mitigates Gall Effects When Susceptible Plants Fail to Elicit Induced Defense

**DOI:** 10.3390/plants13111472

**Published:** 2024-05-26

**Authors:** Janete Ferreira Andrade, Eduardo Soares Calixto, Guilherme Ramos Demetrio, Henrique Venâncio, Marcos Vinicius Meiado, Denise Garcia de Santana, Pablo Cuevas-Reyes, Wanessa Rejane de Almeida, Jean Carlos Santos

**Affiliations:** 1 Department of Systematics and Ecology, Federal University of Paraíba, João Pessoa 58051-900, Paraíba, Brazil; janebiologia@gmail.com; 2 Entomology and Nematology Department, West Florida Research and Education Center, University of Florida, Jay, FL 32565, USA; calixtos.edu@gmail.com; 3 Laboratory of Plant Ecology, U. E. Penedo, Campus Arapiraca, Federal University of Alagoas, Penedo 57200-000, Alagoas, Brazil; guilherme.ferreira@penedo.ufal.br; 4 Graduate Program in Entomology, Faculty of Philosophy, Sciences, and Literature, and Sciences of Ribeirão Preto, University of São Paulo, Ribeirão Preto 14040-901, São Paulo, Brazil; henrivens@gmail.com; 5 Laboratory of Seed Physiology, Biosciences Department, Federal University of Sergipe, Itabaiana 49107-230, Sergipe, Brazil; meiado@academico.ufs.br; 6 Instituto de Ciências Agrárias, Universidade Federal de Uberlândia, Uberlândia 38400-902, Minas Gerais, Brazil; denise.santana@ufu.br; 7 Laboratorio de Ecología de Interacciones Bióticas, Universidad Michoacana de San Nicolás de Hidalgo, Ciudad Universitaria, Morelia 58004, Michoacán, Mexico; pcragalla@gmail.com; 8 Graduate Program in Ecology and Conservation, Federal University of Sergipe, São Cristóvão 49107-230, Sergipe, Brazil; wanereal@yahoo.com.br; 9 Department of Ecology, Federal University of Sergipe, São Cristóvão 49107-230, Sergipe, Brazil

**Keywords:** *Bauhinia brevipes*, plant-induced defense, plant performance, phenotypes, transgenerational success

## Abstract

Variations in plant genotypes and phenotypes are expressed in ways that lead to the development of defensive abilities against herbivory. Induced defenses are mechanisms that affect herbivore insect preferences and performance. We evaluated the performance of resistant and susceptible phenotypes of *Bauhinia brevipes* (Fabaceae) against attacks by the gall-inducing insect *Schizomyia macrocapillata* (Diptera). We hypothesized that there is a positive relationship between resistance to *S. macrocapillata* and host plant performance because resistance can have a high adaptive value. We evaluated plant architecture, nutritional leaf quality, leaf fluctuating asymmetry, and reproductive capacity between phenotypes. Plant performance was evaluated at three ontogenetic stages: seed, seedling, and juvenile. Overall, there were no differences in vegetative and reproductive performance or asymmetry between the resistant and susceptible mature plants. We found no relationship between leaf nutritional quality and resistance to *S. macrocapillata*. Plant performance was consistent across ontogeny for both phenotypes, except for five variables. Contrary to our expectations, the susceptible plants performed equally well or better than the resistant plants, suggesting that tolerance and overcompensation to herbivory in *B. brevipes* may be mediated by induced defense. Our study highlights the importance of multiple layers of plant defense against herbivory, where plant tolerance acts as a secondary barrier in plants susceptible to gall-inducing insects.

## 1. Introduction

Insect–plant interactions represent networks of complex relationships that have been established throughout the coexistence history of both groups [1,2,3]. Insect herbivores are a major component of the world’s biodiversity and are represented by the main trophic interactions of tropical communities [4]. In this context, herbivory is defined as plant consumption by insect herbivores, which is one of the most emblematic ecological processes in natural and anthropized communities [5,6,7]. In particular, herbivory is one of the most ubiquitous and pervasive biotic interactions on the planet [8], and herbivore species may belong to many different functional guilds, such as leaf chewers, branch borers, sap suckers, leaf miners, and gall-inducing insects. This last group of organisms causes hypertrophy and/or hyperplasia of leaf tissue cells in their host plants as a result of gall induction [9,10,11,12]. Gall induction is produced either by saliva injection or other fluid secretions produced by adult insects while laying eggs or by larvae or adult insects inside the plant tissue [13,14,15]. Each gall-inducing insect species has the ability to manipulate the growth and development of plant tissues [16,17] and may also be capable of modifying host nutritional quality and plant secondary metabolites for protection against natural enemies [15,17,18,19,20].

Regardless of the herbivore guild, herbivory has direct effects on plant performance and fitness, as well as indirect effects on ecological processes, such as net primary productivity and nutrient cycling in terrestrial ecosystems [21,22]. The most listed effects of herbivory are deleterious for plants, including individual decreases in growth, reproduction, and survival [23], which influence plant abundance, distribution, and richness [24,25,26].

Understanding the plant defense mechanisms involved in herbivory is a key topic for understanding the ecological importance of plant life history traits and reproductive allocation, which affect the population and communities of plants [22,25]. Life history theory predicts that reproductive success is maximized over time [27], which implies the presence of trade-offs among plant traits, such as defense and reproduction [28]. In this context, herbivores can drive evolutionary changes in plant populations [29]. However, plant responses may vary among plant individuals from the same population [30,31], which has been overlooked in the ecological literature on plant–insect interactions [7]. This is especially true for herbivory caused by gall-inducing insects because of their sedentary feeding habit of larvae, which imposes a strict relationship with their host plants, in which insect development occurs along the galled organ development.

Because insect herbivore occurrence can vary over space and time [32] and plant defenses may be costly [33,34], it is possible that some plant species induce resistance against subsequent herbivore attacks [15,35]. The best documented case is the induction of plant defenses [36,37] that can vary with insect herbivore species as well as with the amount of damaged leaves on the host plants [37,38]. In this way, the plant hypersensitive reaction (HR) arises as a mechanism of plant resistance that has been described in relation to plant damage [39,40,41]. The HR involves morphological and histological alterations that result in the elimination of plant tissues damaged by insect herbivores, subsequently leading to the containment, neutralization, and death of the aggressive agent [39,40]. Under this scenario, the HR has potential significance as a plant defense mechanism against insect herbivore attacks [39,42] and could be particularly prevalent among insect herbivore species closely linked with a particular host plant, such as in the case of gall-inducing insects associated with super hosts [43,44,45,46].

This type of defensive mechanism can have transgenerational effects [47], depending on epigenetic effects [48,49]. Therefore, it is possible to expect the offspring of plants that present higher levels of induced resistance to express reinforced defense traits [50,51]. In this sense, herbivory can drive phenotypic diversity in plant populations [51]. The HR response has already been documented for *Bauhinia brevipes* Vogel (Fabaceae) [43], but no research has evaluated the possible effects of these induced defenses on the performance of the offspring.

We evaluated the plant performance of *B. brevipes* associated with its ability to elicit a HR in response to an attack by *Schizomyia macrocapillata* Maia, 2005 (Diptera: Cecidomyiidae). Our aim was to address the following questions: (1) What are the distinctions between plant individuals capable of eliciting HRs in terms of their vegetative life history traits, reproductive investment, and success rate? (2) And how do different parental phenotypes, specifically susceptible and resistant plants, affect the offspring’s early performance? Considering that nutritional quality can influence plants’ defensive ability [52], we expected that (1) an increase in plant nutritional content would increase their ability to induce HRs against attacks by *S. macrocapillata*. Because the incidence of gall-inducing insects reduces plant fitness [53,54], it can increase environmental stress and be expressed in terms of higher levels of leaf fluctuating asymmetry [55]. We also expected (2) a positive relationship between the capacity to develop HRs and vegetative growth traits, as well as less defended plants, consequently showing higher gall incidence and presenting signs of environmental stress as fluctuating asymmetry. In addition, as the incidence of galls also reduces reproductive success [56], we expected that (3) the ability to develop HRs against *S. macrocapillata* is related to better reproductive performance and influences the development patterns of subsequent generations.

## 2. Results

### 2.1. Hypersensitivity Reaction and Gall-Related Parameters

**Figure 1:**
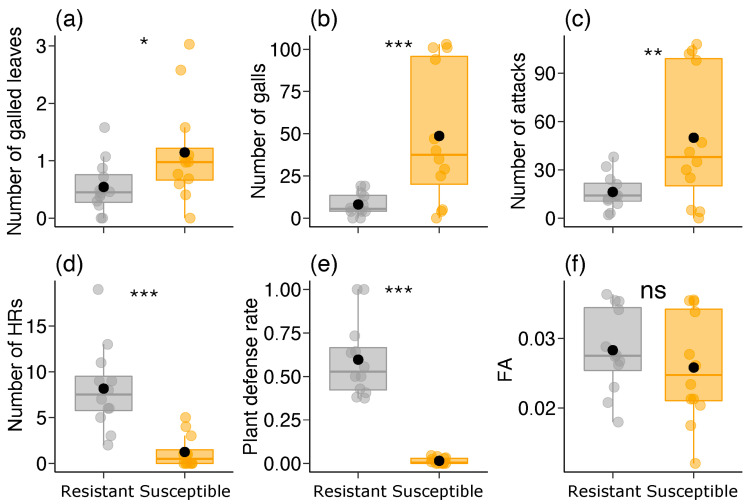
Variation in gall-related parameters [(**a**) number of galled leaves, (**b**) number of galls on leaves, (**c**) number of times the leaf was attacked by adult females, (**d**) number of hypersensitive reactions/HRs in leaves, (**e**) plant defense rate against galling insects, and (**f**) fluctuating asymmetry/FA in leaves] between the two phenotypes (resistant and susceptible) of *Bauhinia brevipes* plants against attacks by *Schizomyia macrocapillata*. Plots show the median and mean (black dot), first and third quartiles, and upper and lower whiskers. See HR and FA explanations in Methods. The small dots represent raw data (n = 12 plants per phenotype). ns—non-significant, * *p* < 0.05, ** *p* < 0.01, *** *p* < 0.001. HR, hypersensitive response; FA, fluctuating asymmetry.

We found significant differences in all the gall-related parameters between the two *B. brevipes* phenotypes (GLM: *p* < 0.03), except fluctuating asymmetry (χ^2^ = 0.759, df = 1, *p* = 0.383) (Table 1; Figure 1). The susceptible phenotype showed higher values for the number of galled leaves, the number of galls, and the number of attacks, while the resistant phenotype showed higher values for the number of HRs (Table 1; Figure 1a–e).

### 2.2. Foliar Nutrients

**Figure 2:**
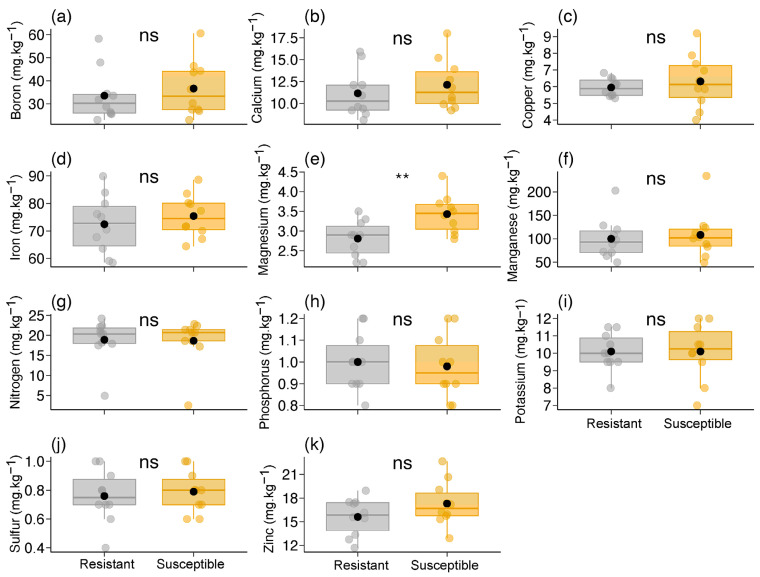
Variation in foliar nutrients [(**a**) boron, (**b**) calcium, (**c**) cooper, (**d**) iron, (**e**) magnesium, (**f**) manganese, (**g**) nitrogen, (**h**) phosphorus, (**i**) potassium, (**j**) sulfur, and (**k**) zinc] between the two phenotypes (resistant and susceptible) of *Bauhinia brevipes* against attacks by *Schizomyia macrocapillata*. Plots show the median and mean (black dot) and first and third quartiles, and upper and lower whisker plots show the median and mean (black dot) and maximum and minimum. The small dots represent raw data (n = 10 plants per phenotype). ns—non-significant, ** *p* < 0.01.

We found no differences in any foliar nutrients between the two *B. brevipes* phenotypes (GLM: *p* > 0.1), except for magnesium (χ^2^ = 8.758, df = 1, *p* = 0.003) (Table 1; Figure 2). The susceptible phenotype had a higher concentration (ca. 1.2× ) of Mg in its leaves compared to the resistant phenotype (Table 1; Figure 2e).

### 2.3. Plant Vegetative and Reproductive Traits

**Figure 3:**
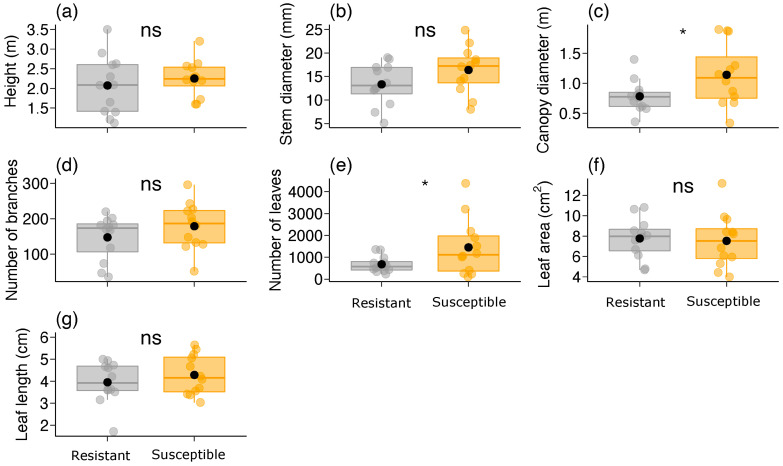
Variation in vegetative plant traits [(**a**) height, (**b**) stem diameter, (**c**) canopy diameter, (**d**) number of branches, (**e**) number of leaves, (**f**) leaf area, and (**g**) leaf length] between the two phenotypes (resistant and susceptible) of *Bauhinia brevipes* against *Schizomyia macrocapillata* attacks. Plots show the median and mean (black dot), first and third quartiles, and upper and lower whiskers. The small dots represent raw data (n = 12 plants per phenotype). ns—non-significant, * *p* < 0.05.

**Figure 4:**
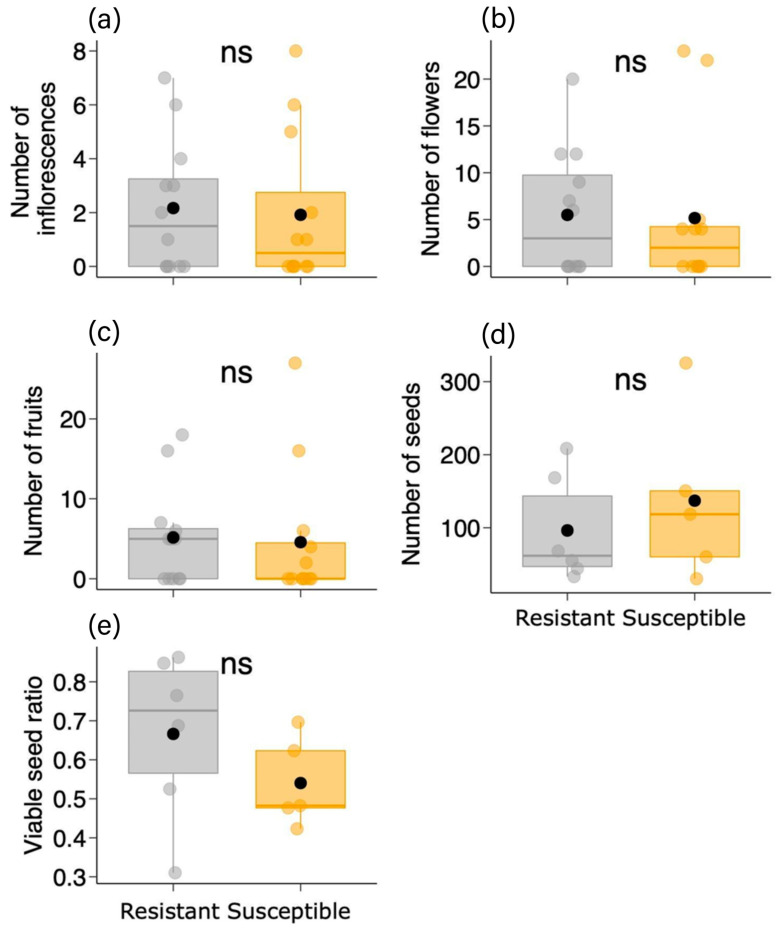
Variation in reproductive plant traits [(**a**) number of inflorescences, (**b**) number of flowers, (**c**) number of fruits, (**d**) number of seeds, and (**e**) viable seed ratio] between the two phenotypes (resistant and susceptible) of *Bauhinia brevipes* against *Schizomyia macrocapillata* attacks. Plots show the median and mean (black dot), first and third quartiles, and upper and lower whiskers. The small dots represent raw data (n = 12 individuals per phenotype). ns—non-significant.

We found no difference in vegetative plant traits between the two *B. brevipes* phenotypes (resistant and susceptible) (GLM: *p* > 0.1), except for canopy diameter (χ^2^ = 4.533, df = 1, *p* = 0.033) and the number of leaves (χ^2^ = 6.013, df = 1, *p* = 0.014) (Table 1; Figure 3). The susceptible phenotype had a larger canopy diameter (ca. 1.5×) and more than twice the number of leaves compared to the resistant phenotype (Table 1; Figure 3c,e). In the case of reproductive plant traits, we did not observe any differences between the two phenotypes (*p* > 0.4) (Table 1; Figure 4).

### 2.4. Germination Rate

**Figure 5:**
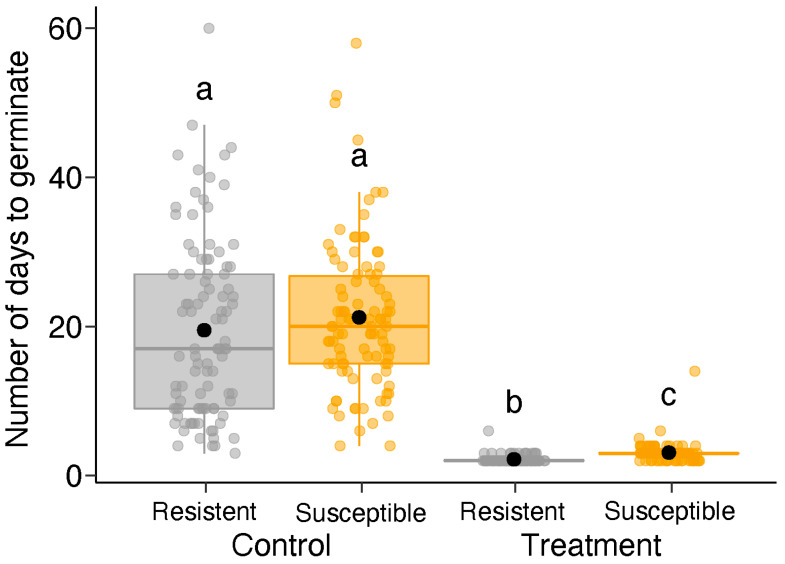
Variation in the number of days to germination between the two phenotypes (resistant and susceptible) and the two treatments (with and without scarification) of *Bauhinia brevipes* plants against *Schizomyia macrocapillata*. Plots show the median and mean (black dot), first and third quartiles, and upper and lower whiskers. The small dots represent the raw data of 385 seeds used in the experiment (n per group: resistant without scarification = 97; susceptible without scarification = 98; resistant with scarification = 93; susceptible without scarification = 97). Different letters indicate significant differences according to Tukey’s test. Control, without scarification; Treatment, with scarification.

Out of 400 seeds, a total of 385 germinated (n per group: resistant without scarification = 97, susceptible without scarification = 98, resistant with scarification = 93, and susceptible without scarification = 97). We found a significant effect of phenotype (GLM: χ^2^ = 7.47, df = 1, *p* = 0.006), scarification treatment (GLM: χ^2^ = 1134.30, df = 1, *p* = 0.001), and the interaction between phenotype and scarification treatment on the number of days taken to germinate (GLM: χ^2^ = 4.080, df = 1, *p* = 0.043; Figure 5). Overall, the non-scarified plants took longer (resistant: 19.4 ± 12.0; susceptible: 21.2 ± 9.9; mean ± SD) to germinate than the scarified plants (resistant: 2.1 ± 0.5; susceptible: 3.0 ± 1.3) (Figure 5). The plant phenotypes from the non-scarified group did not differ from each other, whereas the susceptible phenotype of the scarified plants took a day longer to germinate than the non-scarified plants (Figure 5).

### 2.5. Seedling and Sapling Parameters

**Figure 6:**
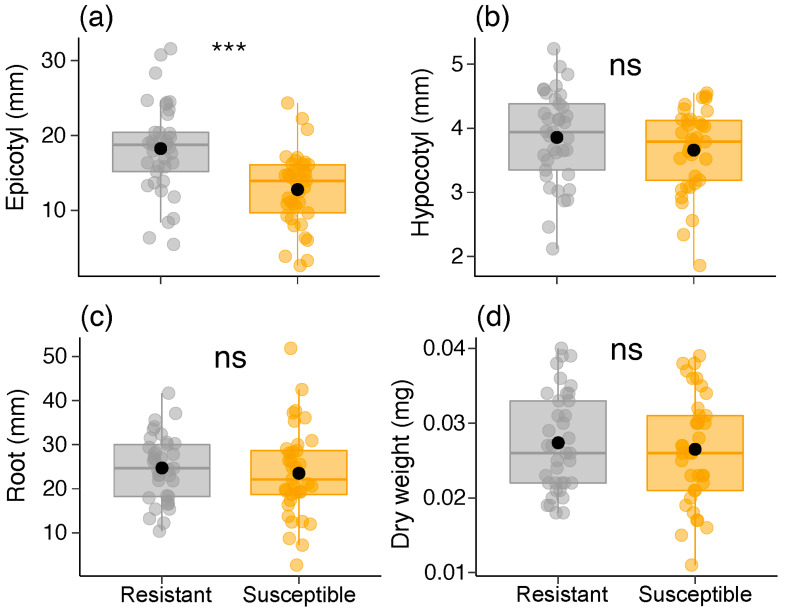
Variation in seedling parameters [(**a**) epicotyl size, (**b**) hypocotyl size, (**c**) root size, and (**d**) dry weight] between the two phenotypes (resistant and susceptible) of *Bauhinia brevipes* against *Schizomyia macrocapillata* attacks. Plots show the median and mean (black dot), first and third quartiles, and upper and lower whiskers. The small dots represent the raw data (n = 40 seedlings per phenotype). ns, non-significant; *** *p* < 0.001.

**Figure 7:**
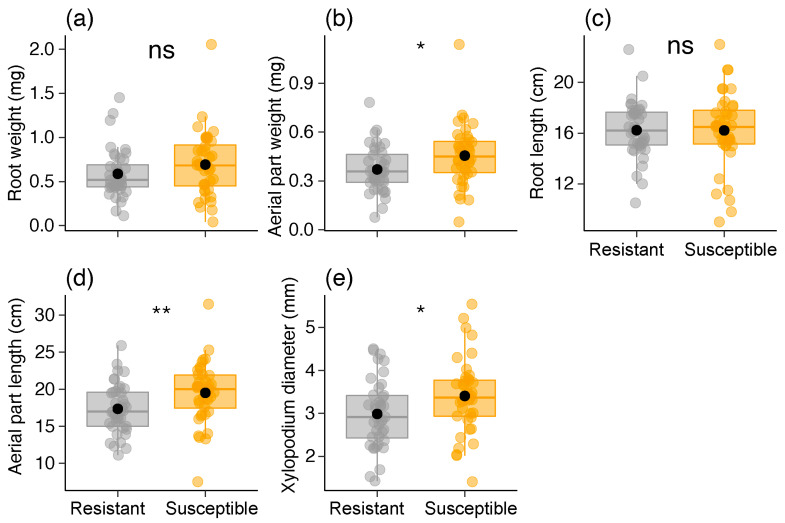
Variation in sapling parameters [(**a**) root weight, (**b**) aerial part weight, (**c**) root length, (**d**) aerial part length, and (**e**) xylopodium diameter] between the two phenotypes (resistant and susceptible) of *Bauhinia brevipes* against *Schizomyia macrocapillata* attacks. Plots show the median and mean (black dot), first and third quartiles, and upper and lower whiskers. The small dots represent the raw data (n = 40 saplings per phenotype). ns—non-significant, * *p* < 0.05, ** *p* < 0.01.

Regarding seedlings, we found differences between the two *B. brevipes* phenotypes only in terms of epicotyl length (χ^2^ = 18.108, df = 1, *p* < 0.001) (Table 1; Figure 6). The resistant phenotype was 50% larger than the susceptible phenotype (Table 1; Figure 6a). Regarding sapling parameters, we did not detect variations in the root weight and length (*p* > 0.1). In contrast, we found variations in the aerial part length and weight, as well as in the xylopodium diameter in the susceptible phenotypes (*p* < 0.024) (Table 1; Figure 7). The results also indicated that the FA levels of the seedlings were similar between the two phenotypes (Table 1).

## 3. Discussion

**Figure 8:**
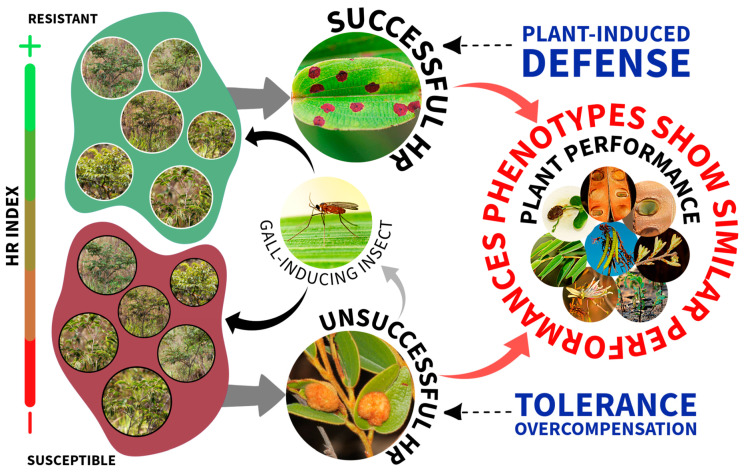
A conceptual scheme of strategies for plant resistance to herbivory in *Bauhinia brevipes* (Fababace). Strategies for plant resistance to herbivory in *B. brevipes* (Fabaceae) involve several mechanisms that aim to reduce the preference and performance of the galling insect *Schizomyia macrocapillata* (Diptera). This is achieved by preventing the formation of galls on the leaves of the resistant phenotype, which can counter galls through induced defense. Susceptible plants are unable to defeat gall-inducing insects and overcome their galls; therefore, they use tolerance and/or overcompensation mechanisms to match or outperform plants with resistant phenotypes. Despite the strong ecological and evolutionary pressures exerted by galls on *B. brevipes* populations, the plants maintain similar fitness owing to their anti-herbivory defenses.

We predicted that the elicited resistance of *B. brevipes* would lead to improved performance among the resistant plants, specifically those capable of developing defenses against gall-inducing insects. Surprisingly, the susceptible phenotypes exhibited similar or greater values for the examined variables, raising the possibility that tolerance to herbivory in *B. brevipes* is favored by induced defense mechanisms (Figure 8). Our results showed significant differences associated with the HR, which was corroborated by the positive relationship detected between the gall-resistant and gall-susceptible phenotypes. The resistant and susceptible phenotypes showed similar reproductive, vegetative, and nutritional performance, except for differences in the magnesium content and the number of leaves. Additionally, we found that the seeds from the susceptible plants took longer to germinate than those from the resistant plants. Similarly, the seedlings from the susceptible plants showed aerial parts with a greater size and biomass, indicating better resource acquisition performance. The seedlings obtained from the susceptible plants also had a larger xylopodium diameter, suggesting a greater ability for resource storage.

Some evidence supports this argument. Initially, it was observed that the fitness of *B. brevipes* did not change with damage and was consistently neutral and on par with that of the resistant plants, which indicates that plants can tolerate injury. Tolerance is the capacity of an organism to thrive and reproduce despite environmental, physical, or biotic stress [57], and plant tolerance has been assessed based on the damage caused by herbivorous insects [58]. Therefore, in evolutionary terms, tolerance is expected to reduce or mitigate fitness losses [58]. Therefore, it is likely that *B. brevipes* employs various strategies to defend itself against herbivory. One such strategy is to induce plant defense responses against insect galls by employing herbivore-induced resistance (expressed as HR in the studied plants) [39,40] and/or tolerance mechanisms [59]. The resistant phenotype exhibits resistance to galls through induced resistance, often referred to as a high HR. In contrast, susceptible plants possess alternative mechanisms for enduring damage. Resistant plants rely primarily on induced resistance as their primary defense strategy, whereas susceptible plants employ a different tolerance mechanism. Although in both phenotypes resistance is induced, in one it is activated in response to galls (resistant plants), whereas in the other it is not (susceptible plants). Subsequently, susceptible plants employ different strategies to tolerate and/or compensate for damage caused by insect galls.

Although the connection between fitness and damage may be positive, the tolerance mechanism may generate responses that could be perceived as positive for the plant. This means that damaged plants may exhibit greater fitness than undamaged ones, a mechanism known as overcompensation [58]. This would explain the results obtained, such as the higher levels of magnesium and the greater number of leaves in susceptible plants. In all cases, the susceptible plants performed better than the resistant plants, which were able to efficiently stop galling insects from developing galls. Galls are drains that negatively impact the fitness of their host plants [60,61,62,63].

However, the susceptible *B. brevipes* plants overcame these impacts and continued to invest more resources in nutrition and vegetative growth, improving the performance of their offspring through greater vegetative growth in terms of the various parameters analyzed in our study. Several host plants show a strong investment of resources in growth and reproduction, which is regarded as an important event in plant genotype adaptation [64,65]. As for the resistant *B. brevipes* plants, once the induced defense was effective, these plants did not manifest any tolerance mechanism, even considering their better performance in only two variables (i.e., number of days to germination and epicotyl size).

Plant performance depends on environmental and biological conditions that influence plant quality and resource allocation. For example, previous studies have shown increases in secondary compound production after simulated herbivory of *B. brevipes* leaves [66]. Additionally, the super host capacity of *B. brevipes* provides evidence that simultaneous multi-taxon attacks have synergistic effects on overall plant performance. This can be explained by a reduction in photosynthetic plant tissue as well as the high cost of defenses [56]. In this way, resistant genotypes could be favored in high herbivory damage scenarios, having a high cost associated with resistance and, therefore, a disadvantage in low herbivory damage scenarios [59,67,68]. Thus, considering that the susceptible plants of *B. brevipes* showed similar performance in comparison with the resistant plants, our results suggest that these plants invest more resources in defense. However, susceptible plants may overcompensate for herbivore damage by allocating resources to aerial part growth, as can be seen through their larger canopy diameter and leaf number, which can be used as estimators of reproductive investment [69].

Macro- and micronutrient concentrations in the leaves were similar when comparing the resistant and susceptible phenotypes of *B. brevipes*. All plants were sampled from a relatively small area with homogeneous soil characteristics, which may explain the low variation in nutrient concentrations in both plant groups. In addition, soil nutritional intake is low in savannah plant species [68], which may lead to similarities in nutritional concentrations between resistant and susceptible plants.

Leaf asymmetry was also similar between the resistant and susceptible phenotypes when the adult plants and offspring of the two groups were compared. In the adults, these results were unexpected, as it was previously shown that *S. macrocapillata* attacks and HR defense were associated with FA levels in *B. brevipes* [55,70]. We assessed the asymmetric response of non-galled and non-necrotic leaves. Therefore, one possible explanation is that the stress response to herbivory and the induced defense found on galled leaves [70] may not be reflected in non-attacked leaves. This hypothesis is reinforced by the absence of a difference in nitrogen content between the two phenotypes since, in leaves, this nutrient is affected by AF level and HRs [55,71]. Moreover, gall attacks did not negatively affect some parameters of non-attacked modules in some plant species [60], a characteristic that may also be associated with asymmetry. In relation to offspring, we found similar AF between the groups despite the increased aerial and xylopodium parameters in the susceptible individuals, which may indicate that transgenerational responses to herbivory were not stressful under our greenhouse conditions. This may have occurred because of the favorable abundance of resources (e.g., soil nutrients, water, and light) and storage (i.e., increased xylopodium diameter) for the development of *B. brevipes*, which improved the overcompensation response of susceptible saplings and did not result in physiological stress [72].

In addition to asymmetry, the offspring of the resistant and susceptible phenotypes showed some performance similarities. However, the seedlings from the susceptible plants needed more time to germinate and had higher aerial part growth rates. This fact suggests the absence of a transgenerational effect, as one could expect susceptible plant offspring to present higher investment in defense [47,48]. In this sense, we suggest that the higher growth and resource storage ability shown by the susceptible plant offspring is linked to the very low rate of attack by *S. macrocapillata* suffered by the parental plants, which did not induce resistance against this herbivore species. Notwithstanding the absence of genetic regulation of plant genotypes, it is impossible to achieve precise control of transgenerational outcomes. Therefore, additional experimental and genetic research is required to address this issue.

## 4. Conclusions

Our study demonstrated that the degree of performance exhibited by susceptible and resistant phenotypes could be explained by the fact that susceptible plants possess the ability to tolerate or compensate for the harm caused by *S. macrocapillata*. However, our research also revealed the intricate manner in which different defense mechanisms interact in *B. brevipes* to protect against, alleviate, or even surpass the damage inflicted by gall-inducing insects. Notably, these effects persisted across generations. It will be crucial to investigate the genetic underpinnings of these phenotypes and explore the potential involvement of additional defense mechanisms.

## 5. Material and Methods

### 5.1. Study Area

**Figure 9:**
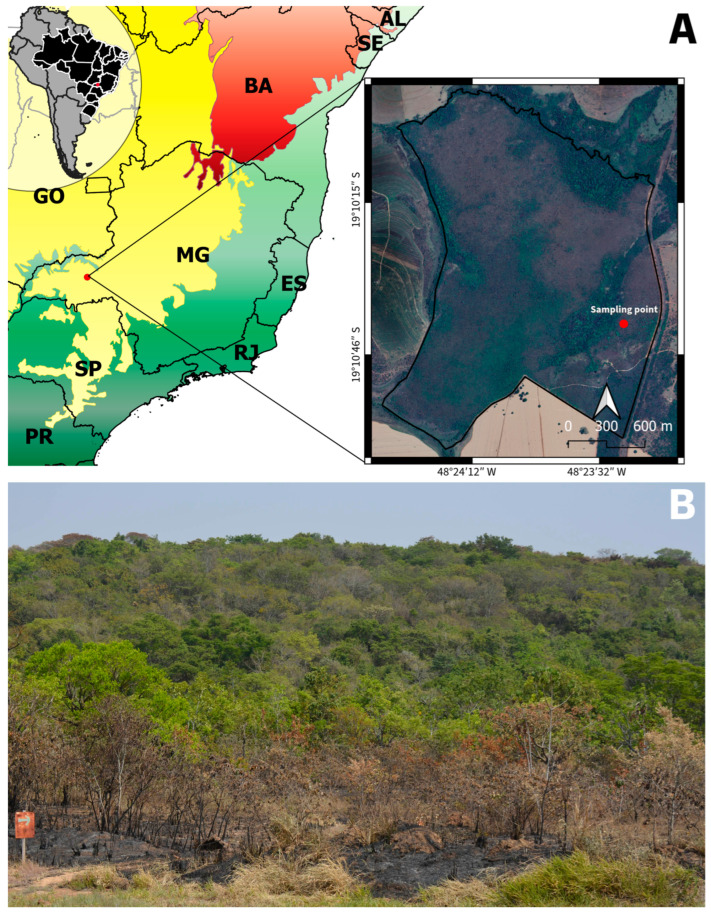
Study area: (**A**) geographical location and (**B**) panoramic view of the vegetation. Credit for map: A. B. S. Farias.

The study was conducted at the Panga Ecological Station, a protected area located 30 km south of Uberlândia, State of Minas Gerais, Brazil (19°10′46″ S and 48°23′32″ W) (Figure 9). This region is characterized by a subtropical climate with two well-defined seasons: a dry winter from May to September and a rainy summer from October to April [73,74]. The average annual temperature is approximately 22 °C, and the total precipitation is 1650 mm per year. The soils were primarily red latosols, but they varied from moderate to strongly acidic. The vegetation comprises different areas of the Brazilian Cerrado (savanna) and forest formations [75].

### 5.2. Study System

*Bauhinia brevipes* is a shrub species that grows up to three meters tall and is widely distributed around the states of Bahia, Goiás, Mato Grosso, Mato Grosso do Sul, Minas Gerais, Piauí, Rondônia, São Paulo, and Tocantins [76]. It is a deciduous species that loses leaves between May and August. New leaves sprout at the beginning of October, concurrent with the beginning of the rainy season. The flowering period begins in May, peaks in July, and ends in September [77].

*Schizomyia macrocapillata* Maia, 2005 (Diptera: Cecidomyiidae) induces galls of red color, covered by trichomes, formed on the adaxial leaf surface. Galls occur as single or coalescent groups [78]. Oviposition occurs at the beginning of the rainy season (October), when leaves are young and still unfolded [79]. Gall development occurs from October to March, when eggs are laid. Gall survivorship is low, with more than 90% of the larvae killed by hypersensitive responses [80]. We chose *S. macrocapillata* as the test insect species because it is one of the main gall-inducing species of *B. brevipes* and the only one that elicits hypersensitivity responses in this host species [80]. In our investigation, we chose a sample of 42 plants that were examined in the field, and all the measurements and evaluations were conducted using the same set of plants.

### 5.3. Hypersensitivity Reaction and Gall-Related Parameters

**Figure 10:**
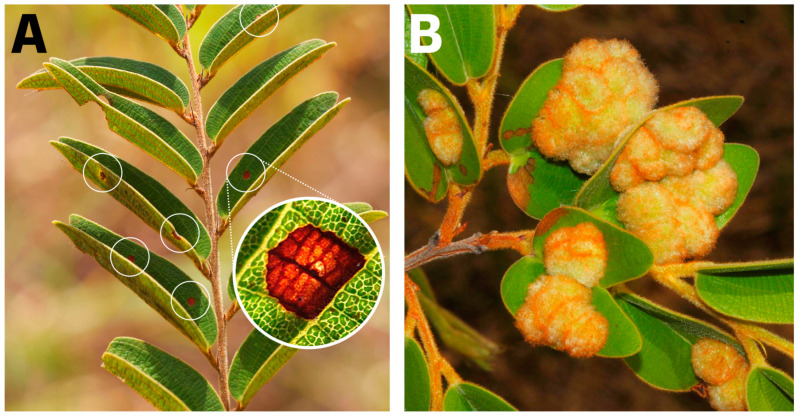
Comparison of two phenotypes (resistant and susceptible) of *Bauhinia brevipes* (Fabaceae) plants against *Schizomyia macrocapillata* (Diptera: Cecidomyiidae). (**A**) Resistant phenotype, characterized by several signs of hypersensitivity reactions (in detail) as circular yellowish-brown spots on the leaf surfaces (white circles), which occur because of oviposition by gall-inducing insects. (**B**) Susceptible phenotype, characterized by several galls of *S. macrocapillata* on leaves.

**Figure 11:**
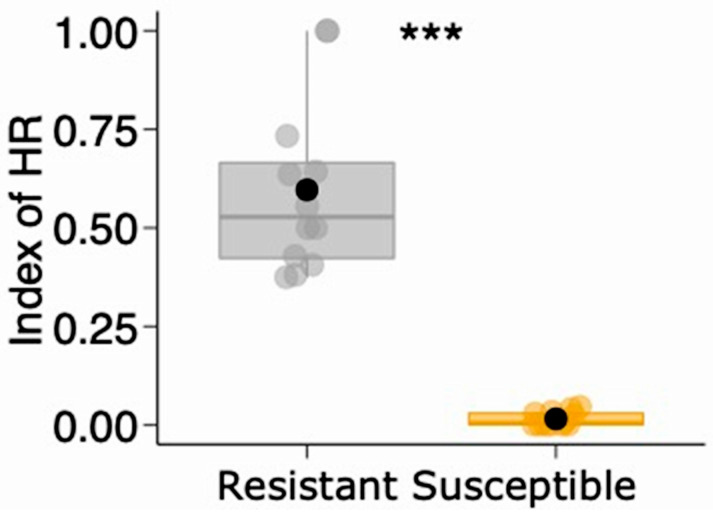
Variation in hypersensitive reaction index (HRI) between the two phenotypes (resistant and susceptible) of *Bauhinia brevipes* plants against the attack of *Schizomyia macrocapillata*. Plots show the median and mean (black dot), first and third quartiles, and upper and lower whiskers. The small dots represent raw data (n = 12 plants per phenotype). *** GLM: χ^2^ = 29.084, *p* < 0.001.

We characterized the hypersensitivity reaction (HR) as the presence of circular yellowish-brown spots on the leaf surfaces that occur as a result of an attempt at oviposition by endogenous insects such as the gall-inducing *S. macrocapillata* [42,81]. In March 2013, we evaluated the number of HR inductions in *B. brevipes* individuals that were at least 10 m apart from each other in an adult population (N = 42 plants) to identify two phenotypic groups of plants. For that, we calculated the Hypersensitive Reaction Index (HRI), which was calculated as follows: “HRI = number of HR/(number of HR + number of galls) × 100”. Plants with a HRI *>* 0.1 were classified as resistant, and plants with a HRI ≤ 0.1 were classified as susceptible. This HRI allowed us to separate 12 individuals with higher HR frequencies and 12 individuals with lower HR frequencies and categorize them into two phenotypes: “resistant” (high frequency of HRs and low number of *S. macrocapillata* galls) or “susceptible” (low frequency of HRs and high number of *S. macrocapillata* galls) (Figure 10). Intermediate individuals were not considered in the analyses (N = 18) and were, therefore, excluded. To validate this approach, we compared the HRI between phenotypes (resistant and susceptible) using a Generalized Linear Model (GLM) with a beta distribution, followed by the likelihood ratio test. Indeed, the resistant plants showed a significantly higher HRI (0.59 ± 0.21; mean ± SD) than the susceptible plants (0.01 ± 0.01) (GLM: χ^2^ = 29.084, *p* < 0.001; Figure 11), proving our hypothesis that there are two distinct phenotypes based on induced plant resistance in *B. brevipes*. Previous studies have also demonstrated that natural populations of *B. brevipes* exhibit phenotypic responses to induced defense against galling insects [43,82].

### 5.4. Nutritional Content

To assess if a plant’s nutritional content increases its ability to develop HR against *S. macrocapillata* attacks, the nutrient content of the leaf tissues of the 24 previously selected plant individuals was analyzed (N = 12 for the resistant phenotype and N = 12 for the susceptible phenotype). For this, in December 2013, we randomly collected 20 fully expanded and healthy leaves from the middle canopy of each plant of the two phenotypes (N = 240 leaves per phenotype), dehydrated all leaves at 70 °C for 48 h, and sub-sampled 10 g of dry biomass per plant for chemical characterization. We used the methods suggested by Santos et al. [55] to determine the following mineral contents: nitrogen (N), phosphorus (P), potassium (K), calcium (Ca), magnesium (Mg), and sulfur (S), as macronutrients, and boron (B), copper (Cu), iron (Fe), manganese (Mn), and zinc (Zn). All nutrients were analyzed at the Laboratory of Soils of Universidade Federal de Uberlândia (Uberlândia, Minas Gerais, Brazil) in December 2013. The nitrogen content was analyzed by flow injection analysis; phosphorus was determined colorimetrically; potassium was determined using an air–acetylene flame spectrophotometer; calcium, magnesium, sulfur, iron, copper, and manganese were measured by atomic absorption spectrophotometry; and boron was determined using the hot-water method.

### 5.5. Vegetative Plant Traits and Fluctuating Asymmetry

To evaluate the relationship between the capacity to induce HR and the vegetative traits, as well as signs of environmental stress such as fluctuating asymmetry, we recorded and compared a set of traits from the resistant and susceptible plants, such as height and canopy diameter, using a hard tape and counted the absolute number of branches and leaves on a branch, estimating the total number of leaves per plant. Finally, in December 2013, we randomly collected 20 fully expanded leaves from each plant class, and at the lab, we scanned all of them on a white background with a known scale. Thus, we evaluated leaf length and area and fluctuating asymmetry (FA) measurements using ImageJ software (version 1.54) [83].

### 5.6. Fluctuating Asymmetry Analyses

To assess leaf FA (mm), we measured the width of all leaves (N = 480 leaves) on both the right (*Rw*) and left sides (*Lw*) from the midrib (man vein) to the leaf edge once *B. brevipes* leaves were bi-lobed (see Santos et al., 2013). FA was calculated as the absolute mean difference between the width of the right and left halves of the leaves: FA = [(∑ |(*Rw* − *Lw*)|/*n*], where n represents the number of leaves measured [84].

We tested the accuracy of the measurements previously performed by sampling (randomly) a subset of 50 leaves and measuring them again in the same manner. We compared these measurements with the original *Rw* and *Lw* measurements and estimated the Falconer’s Index of Repeatability (IR) [85]. The repeatability of the measures is crucial to evaluating whether they have been taken correctly and to discarding errors [55]. We performed a two-way mixed-model ANOVA to determine whether the between-side variation was significantly larger than the measurement error [86]. The measurement errors were irrelevant (IR = 1; *F*_1,49_ = 11.3961; *p* < 0.0001), indicating that the leaves were correctly measured [55].

### 5.7. Reproductive Performance beyond a Generation

To evaluate whether the ability to induce HR against *S. macrocapillata* influences its reproductive performance as well as the development patterns of the following generations (i.e., seeds, seedlings, and saplings), we recorded (1) the absolute number of inflorescences, (2) flowers, and (3) fruits and (4) the average number of seeds per fruit in each plant (in the dry season, between July and October of 2014). Before the seed dispersal period, we carefully bagged all fruits during the fruiting period (September 2014) to ensure the future collection of seeds. Finally, we visually evaluated (5) seed viability by considering non-viable immature and preyed seeds. According to their fitness, the plants were separated into female and male groups, as suggested by Agrawal [56]. The female reproductive performance index was calculated by multiplying the seed number by the mean seed mass [56]. To estimate the male reproductive performance, we calculated the number of fruits/(number of fruits + mean number of bud flowers) to deduce the proportion of bud flowers that were fertilized.

All fruits were carefully bagged during the fruiting period to ensure seed collection. This plant species exhibits explosive seed dispersal [76]. When all fruits were opened, we excluded immature and preyed seeds from the total. The remaining seeds were grouped according to the parental plant phenotype (resistant or susceptible). We broke seed dormancy through the mechanical scarification of seeds with sandpaper. We evaluated the performance of offspring from resistant and susceptible plants in three different phases of the germination process: the seed stage, seedlings, and saplings, as detailed below.

(1). Germination rate

We conducted experimental seed germination to evaluate whether the seeds of the resistant plants would exhibit higher performance than those of the susceptible plants (October to December 2014). In the laboratory, we used 400 seeds from plants of both phenotypes (i.e., resistant and susceptible). In October 2014, the surfaces of all seeds were sterilized by immersion for 10 min in sodium hypochlorite (1 mL per 100 mL of distilled water). After that, we separated 200 seeds per phenotype, of which 100 received treatment for dormancy breaking (scarified seeds) and 100 did not receive any treatment (control). To break the dormancy of the seeds, we performed mechanical scarification, which consists of sanding the upper side of seeds (to avoid damage to the embryo) and sterilizing with 0.05% sodium hypochlorite solution for 5 min before scarification and sterilization for 3 min after scarification [87]. We distributed seeds from both treatments (scarified seeds and control) into plastic gerbox-type boxes to germinate fine-grained vermiculite (~160 mL) at a depth of 1 cm and moistened them in distilled water up to the field capacity (~80 mL). The boxes with seeds were randomly arranged and incubated (in a BOD incubator) at 25 °C in a 12 h light/12 h dark photoperiod under fluorescent light (Santana et al., 2016 [87]). We performed the assessment daily (and at the same time) from sowing by counting the number of seeds with primary root protrusion (germination) and removing dead seeds [88].

(2). Seedlings parameters

To evaluate the morphological differences in the development of *B. brevipes* seedlings, we conducted an alternative seed germination procedure in October 2014. We selected another 40 seeds from the resistant and susceptible plants (N = 80 seeds), which were subjected to the same sterilization procedure as in the previous experiment and dormancy breaking (there was no control treatment in this step). We alternately arranged seeds on two paper towels saturated with distilled water, covered them with two more sheets, and then rolled them up [89]. These rolls were placed in plastic bags and incubated in a BOD incubator at 25 °C under continuous white fluorescent light for 10 days. After this period, in November 2014, we assessed the length (cm) of the epicotyl, hypocotyl, and root, then separated and dried the above- and belowground parts at 80 °C for 24 h and evaluated the dry mass (g) of the seedlings [90].

(3). Sapling traits

To evaluate the performance (investment in growth) and stress of saplings from the resistant and susceptible plants of *B. brevipes*, we analyzed the development of 40 saplings from the resistant and susceptible plants (N = 80). The seeds were distributed in vertical tubes with a mix of commercial substrate (Terra do Campo^®^, Brazil) and vermiculite (Mogifertil^®^, Brazil) (110 cm^3^) in plastic brackets (620 × 420 × 16 mm). The saplings were cultivated, between November 2014 and February 2015, in a greenhouse under natural light conditions, with temperatures ranging from 20 °C to 40 °C and intermittent irrigation (at 12 h intervals for 5 min). After 90 days, we removed the saplings, packed them in properly labeled paper bags, and then separated and dried the aboveground and belowground parts at 70 °C for 48 h. We recorded stem height (cm), dry mass (g), root length (cm), dry mass (g), root/shoot biomass ratio, xylopodium diameter (cm), and leaf area (cm^2^) digitally using Image J software (version 1.54) [62]. Moreover, we randomly selected three fully expanded leaves per individual to evaluate the FA (mm) of juveniles (N leaves per group = 120, N total = 240). Fluctuating asymmetry measurements were taken as the linear distance from the midrib to the right and leaf margin of each leaf, following the previously described method. Asymmetry index 1 (i.e., FA = [(∑ |(*Rw* − *Lw*)|/*n*]) was calculated again to sample the asymmetry of each plant [91].

## 6. Data Analysis

Statistical analyses were performed using the R Environment (version 4.0.0) [92]. Model fit was checked by analyzing the fitted versus residual values plot, the distribution of residuals in a QQ plot, the histogram of residuals, and by using the “DHARMa” package [93], in which we created scaled residuals through simulation from the fitted model. Next, we used a parametric bootstrap (250 randomizations) to compare the observed residuals against the refit residuals, which is indicated for testing overdispersion.

To verify whether there were differences in plant vegetative and reproductive traits, gall-related parameters, plant nutrient content, and seedling and sapling parameters between the two phenotypes (resistant and susceptible) of *B. brevipes*, we performed different GLMs followed by the likelihood-ratio test using the packages ‘stats’ [92] and ‘car’ [94], respectively, for each response variable (Table 1). Each model was fitted to the best data distribution. We used a negative binomial distribution to control for overdispersion in count data.

To evaluate the number of days taken for seeds to germinate between the *B. brevipes* phenotypes and treatments (seed scarification), we used a multifactorial GLM, where the number of days to germinate was considered as the response variable, and the interaction between phenotypes and treatments (phenotype × treatment) was used as the explanatory variable.

Although we mentioned the statistical analyses of seedlings and saplings before germination rate, we wrote our results based on plant ontogeny; that is, first, we described the germination rate and then the seedling and sapling results.

## Figures and Tables

**Table 1:** Model statistics comparing the performances (resistant and susceptible) of two phenotypes of *Bauhinia brevipes* against *Schizomyia macrocapillata* (Cecidomyiidae). Significant *p*-values are shown in bold.

Analysis	Response Variable	Distribution	Likelihood Ratio Test	*p*-Value	Resistant (Mean ± SD)	Susceptible (Mean ± SD)
Gall-related parameters	Number of galled leaves (mean)	Gaussian	4.474	**0.034**	0.54 ± 0.45	1.14 ± 0.87
	Number of galls	Negative Binomial	15.908	**0.001**	8.08 ± 6.77	48.6 ± 40.4
	Number of attacks	Negative Binomial	8.564	**0.003**	16.2 ± 10.8	49.9 ± 41.8
	Number of HRs	Negative Binomial	33.857	**0.001**	8.16 ± 4.62	1.25 ± 1.76
	Fluctuating asymmetry (mm)	Gaussian	0.759	0.383	0.02 ± 0.01	0.02 ± 0.01
Foliar nutrients	Boron (mg·kg^−1^)	Gaussian	0.362	0.547	33.5 ± 11.1	36.6 ± 11.8
	Calcium (mg·kg^−1^)	Gaussian	0.605	0.436	11.1 ± 2.71	12.1 ± 2.85
	Copper (mg·kg^−1^)	Gaussian	0.470	0.492	5.9 ± 0.5	6.3 ± 1.5
	Iron (mg·kg^−1^)	Gaussian	0.527	0.467	72.4 ± 10.4	75.4 ± 7.6
	Magnesium (mg·kg^−1^)	Gaussian	8.758	**0.003**	2.8 ± 0.4	3.4 ± 0.4
	Manganese (mg·kg^−1^)	Gaussian	0.154	0.694	99.9 ± 44.0	108.3 ± 50.8
	Nitrogen (mg·kg^−1^)	Gaussian	0.009	0.921	18.8 ± 5.3	18.6 ± 5.9
	Phosphorus (mg·kg^−1^)	Gaussian	0.101	0.750	1.0 ± 0.1	0.9 ± 0.1
	Potassium (mg·kg^−1^)	Gaussian	0.001	0.99	10.1 ± 1.0	10.1 ± 1.6
	Sulfur (mg·kg^−1^)	Gaussian	0.164	0.685	0.76 ± 0.1	0.79 ± 0.1
	Zinc (mg·kg^−1^)	Gaussian	2.109	0.146	15.6 ± 2.3	17.2 ± 2.8
Vegetative plant traits	Stem diameter (mm)	Gaussian	2.519	0.112	13.3 ± 4.45	16.3 ± 4.93
	Canopy diameter (m)	Gaussian	4.533	**0.033**	0.78 ± 0.26	1.14 ± 0.51
	Number of branches	Negative Binomial	1.097	0.294	147.5 ± 62.8	179.0 ± 65.8
	Number of leaves	Negative Binomial	6.013	**0.014**	679.1 ± 373.4	1452 ± 1300
	Leaf area (cm^2^)	Gaussian	0.063	0.801	7.77 ± 1.96	7.53 ± 2.63
	Leaf length (cm)	Gaussian	0.792	0.373	3.95 ± 0.94	4.28 ± 0.90
Reproductive plant traits	Number of inflorescences	Negative Binomial	0.039	0.843	2.16 ± 2.48	1.91 ± 2.81
	Number of flowers	Negative Binomial	0.005	0.938	5.50 ± 6.68	5.16 ± 8.34
	Number of fruits	Negative Binomial	0.021	0.882	5.16 ± 6.17	4.58 ± 8.46
	Number of seeds	Negative Binomial	0.678	0.410	92.0 ± 73.3	132.6 ± 115.3
*Seedling parameters*	Viable seed ratio	Beta	1.965	0.160	0.66 ± 0.21	0.54 ± 0.11
	Epicotyl (mm)	Gaussian	18.108	**0.001**	18.2 ± 6.0	12.7 ± 4.9
	Hypocotyl (mm)	Gaussian	1.573	0.209	3.8 ± 0.7	3.6 ± 0.6
	Root (mm)	Gaussian	0.335	0.562	24.7 ± 7.4	23.5 ± 10.1
	Dry weight (mg)	Gaussian	0.282	0.595	0.02 ± 0.01	0.02 ± 0.01
Sapling parameters	Root weight (mg)	Gaussian	2.096	0.147	0.58 ± 0.2	0.69 ± 0.3
	Aerial part weight (mg)	Gaussian	5.407	**0.020**	0.37 ± 0.13	0.45 ± 0.18
	Root length (cm)	Gamma	0.001	0.976	16.2 ± 2.2	16.2 ± 2.9
	Aerial part length (cm)	Gaussian	6.813	**0.009**	17.3 ± 3.3	19.5 ± 4.0
	Xylopodium diameter (mm)	Gaussian	5.081	**0.024**	2.9 ± 0.7	3.4 ± 0.8
	Fluctuating asymmetry (mm)	Gaussian	3.316	0.068	0.15 ± 0.07	0.19 ± 0.01

## Data Availability

All relevant data are available in the manuscript.
